# Post-infectious myositis ossificans in medial, lateral pterygoid muscles: A case report and review of the literature

**DOI:** 10.3892/ol.2014.2710

**Published:** 2014-11-19

**Authors:** QIAN JIANG, MIN-JIE CHEN, CHI YANG, YA-TING QIU, ZHEN TIAN, ZHI-YUAN ZHANG, WEI-LIU QIU

**Affiliations:** Department of Oral and Maxillofacial Surgery, Ninth People’s Hospital, Shanghai Jiao Tong University School of Medicine, Shanghai Key Laboratory of Stomatology, Shanghai 200011, P.R. China

**Keywords:** myositis ossificans, infection, pterygoid muscle, computed tomography

## Abstract

Myositis ossificans (MO) is a disease where heterotropic bone forms within a muscle or other type of soft tissue. MO is classified into two groups, MO progressiva and post-traumatic MO. It rarely occurs in the masticatory muscles and thus, only 20 cases involving the masticatory muscles have been reported since 2001. The majority of the reported cases occurred due to trauma, repeated injury or surgical manipulation. However, in a small number of cases, no specific traumatic event was identified as the cause of MO. To the best of our knowledge, this is the first case of post-infectious MO to be reported in the medial and lateral pterygoid muscles.

## Introduction

Myositis ossificans (MO) is a disease where the formation of heterotropic bone occurs within a muscle or other type of soft tissue (1). MO is classified into two groups, MO progressiva (MOP) and post-traumatic MO (PTMO) (2). MOP is an autosomal dominant disease observed within families in which multiple heterotopic ossifications develop systemically in various muscles, fascia, tendons and ligaments of the body (2,3). PTMO is characterized by heterotopic bone formation within muscle tissue as a result of a single or repetitive injury (2,4). PTMO is frequently reported in the orthopedic literature and is prevalent in the quadriceps femoris and brachialis anticus, where there is high risk for injury. However, MO is rare in the masticatory muscles. Only 20 cases of MO in the masticatory muscles were identified during a review of the literature (since 2001), which was conducted in the present study; 16 cases were associated with an evident traumatic cause and were diagnosed definitively as PTMO.

In the present study, a rare case of MO in the medial and lateral pterygoid muscles that was caused by odontogenic infection is presented, which was diagnosed as post-infectious MO (PIMO). To the best of our knowledge, this is the first case of PIMO in multiple masticatory muscles to be reported in the English literature. Written informed consent was obtained from the patient.

## Case report

In January 2010, a 42-year-old female was referred to the Department of Oral and Maxillofacial Surgery, Ninth People’s Hospital (Shanghai, China) with a complaint of the progressive, painless limitation of mouth opening for three years. The patient had no history of evident trauma, however, had experienced pain in the right upper jaw for approximately three years, for which the patient had not received any endodontic or periodontal treatment. In addition, the patient had experienced weakness when biting and chewing, which had endured for more than two years. The patient was administered with non-steroidal anti-inflammatory agents by The First Hospital of Jiaxing (Jiaxing, China), however, no clinical improvement was observed.

Physical examination revealed that the patient was well-nourished and demonstrated no evidence of developmental abnormalities. No facial asymmetry was apparent and the maximal incisal opening at presentation was 2 mm. Despite the limited range of motion, the patient reported no associated pain or changes in the occlusion. On palpation the masseter and temporalis muscles were normal. The lymph nodes (submandibular and deep cervical) were nonpalpable and nontender. Intraorally, the right maxillary third molar residual root and missing mandibular central incisors were examined. The patient’s dental hygiene was poor, however, the oral mucosa and tongue appeared to be healthy.

**Figure 1:**
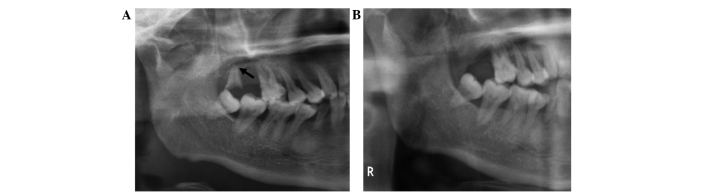
(A) Preoperative panoramic radiography shows the periapical lesions of the right maxillary third molar residual root (shown by the black arrow). (B) Postoperative panoramic radiography demonstrates the extraction of the residual root.

**Figure 2:**
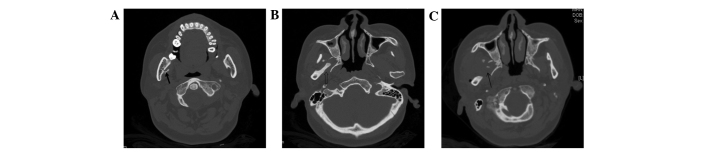
(A) Preoperative computed tomography (CT) scan demonstrating calcification in the right medial pterygoid muscle (solid arrow). (B) Preoperative axial CT showing calcification in the right lateral pterygoid muscle (arrow outline); (C) Postoperative CT scan demonstrating that the ossification was excised (arrow).

**Figure 3:**
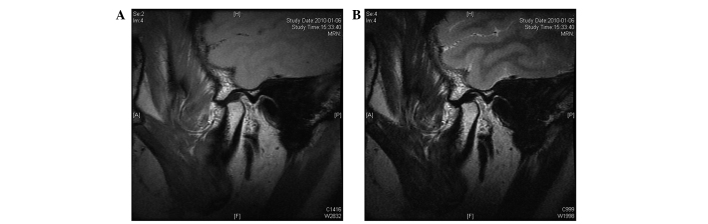
Preoperative magnetic resonance imaging (MRI) examination. Preoperative MRI reveals normal anatomic structures between the articular disk and the condyler in the (A) open and (B) closed jaw positions.

The patient’s general medical status was normal and the laboratory tests, including serum calcium (2.23 mmol/l; normal range, 2.08–2.65 mmol/l) and phosphorus levels (1.46 mmol/l; normal range, 0.78–1.65 mmol/l), were within the normal limits. A panoramic radiograph (Fig. 1) showed chronic periapical lesions of the right maxillary third molar residual root (Fig. 1A). Computed tomography (CT) scans (Fig. 2) revealed heterotopic bone formation in the right lateral pterygoid and medial pterygoid muscles (Fig. 2A and B). Normal anatomic structures between the articular disk and the condyle were observed on magnetic resonance imaging (MRI; Fig. 3A and B). Accounting for the medical history and clinicoradiological results, the patient was diagnosed with MO in the right lateral and medial pterygoid muscles.

**Figure 4:**
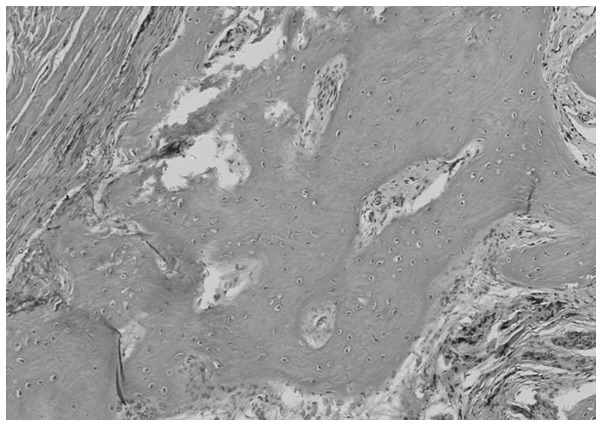
Microphotograph from the pathologic examination of the surgical specimen showing the characteristic features of connective tissue hyperplasia (osteoid and bone). Evident inflammatory cell invasion was observed surrounding the osseous tissue (stain, hematoxylin and eosin; magnification, ×100).

Surgical excision was performed under general anesthesia and, following fiber optic assisted intubation, access to the mandibular was achieved via a preauricular incision, which extended to the temporal region and exposed the condylar process and sigmoid notch, as well as the coronoid process. To remove the calcification in the lateral pterygoid muscle and reduce the tension on the mandible caused by the temporalis, a right coronoidectomy was performed using a reciprocating saw. The majority of the calcified and fibrotic fibers of the upper head of the right lateral pterygoid muscle were removed. An additional intraoral approach to the medial aspect of the mandibular was adopted via a mucoperiosteal incision of the retromolar area to reveal and access the calcified medial pterygoid muscle. A pedicled buccal fat pad (BFP) flap was used to fill the dead space. A calcified mass was identified and subsequently excised. In addition, the masseter attachment was stripped from the ramus of the mandible to weaken the contractile force of the masseter muscle and mouth opening of 40 mm was achieved intraoperatively. The right maxillary third molar residual root was removed. The wound was closed in layers following the achievement of complete hemostasis. The healing period was uneventful and a postoperative panoramic radiograph and a CT scan were performed four days following surgery, which revealed that the ossification had been excised (Figs. 1B and 2C). Histopathology of the excised tissue specimens (Fig. 4) identified the novel formation of bone and osteoid within the muscle fibers. Physical therapy was initiated in the immediate postoperative period using suitable analgesics (200 mg celecoxib, twice a week, for one week) and was continued following discharge from the hospital. Maximum spontaneous mouth opening of 30 mm was achieved seven days following surgery.

The patient was followed up for a total of 36 months postoperatively. At present, the patient exhibits a stable interincisal opening of 25 mm.

## Discussion

MO presenting in the masticatory muscles is rare; a review of the literature, which was conducted in the present study identified only 20 cases reported since 2001. The results of the literature review are presented in Tables I–III. The mean patient age was 36.75 years (range, 18–68 years). Nineteen of the 20 patients initially attended the hospital presenting with restricted mouth opening. Of the 20 patients, 16 were diagnosed with PTMO as a result of facial trauma (4–10), local infiltration of anesthetics (1,11–15), dental surgery (16,17) or absolute alcohol injection (18). However, the remaining four patients had no evident history of trauma, tooth extraction or infection (2,19,20,22). Among the patients diagnosed with PTMO, males predominated (ratio of males to females, 11:5), which may be attributed to the fact that males are more likely to be subject to trauma in daily life (10). The region where PTMO most frequently occurred was the medial pterygoid muscles, which was often caused by a local anesthesia injection, followed by the application of external force directly to the temporalis and masseter muscles. The present review of the literature revealed that out of the 20 cases observed, the surgical management of one case (14) was performed at the early stage of PTMO in the temporalis without the observation of calcification, however, in the other cases, surgery was conducted when trismus occurred and calcification was identified via CT. In addition, of the 17 patients that were followed up, 10 patients were continued with the follow-up for more than one year and four patients exhibited recurrence subsequent to the first surgical treatment.

The exact mechanism for the pathogenesis of MO remains unclear, however, trauma is considered to be the inciting event. According to the literature, a signal, such as a bone morphogenetic protein (BMP) signal from the site of injury, may induce mesenchymal cells to differentiate into osteoblasts or chondroblasts, given the appropriate environment (23,24). In the field of stomatology, odontogenic infection is a common condition when accompanied by trauma. In the present case, the smaller, superficial head of the medial pterygoid muscle, which originates from the maxillary tuberosity was proximal to the maxillary third molar. Therefore, apical periodontitis of the third maxillary molar may have spread toward the medial pterygoid muscles. Furthermore, infections in the medial pterygoid muscles may have spread toward the lateral pterygoid muscles through potential fascial spaces containing loose connective tissue. Long-term, low-grade inflammation may have stimulated the appropriate signaling agents, such as BMP, to induce heterotopic bone formation. Thus, it is hypothesized in the present report that infection and trauma exhibit an equally important role in the pathogenesis of MO in the masticatory muscles. Therefore, the present case was diagnosed as PIMO.

Ossification, the symptom of MO, may be observed by diagnostic imaging tests a minimum of 2–5 weeks subsequent to injury (25–27). After eliminating temporomandibular joint disease using MRI, CT and three-dimensional CT scans are considered to be particularly efficacious investigative tools in the oral and maxillofacial region. These imaging techniques aid with identifying the exact location and shape of the ossification, as well as establishing the association between the lesion and surrounding tissues, which is important for surgical treatment. Although a panoramic radiograph may not be effective for determining the exact extent of the lesion, due to the superimposition of the cranial bones, it may aid with the identification of odontogenic infection foci. Bone scans and ultrasound may also be used, however, are rarely applied for the craniofacial region (28).

Treatment of PTMO and PIMO usually includes surgical excision of the calcification and the surrounding muscles. Patients with MO of the temporalis or masseter area often undergo a coronoidectomy and the excision of the involved calcified muscles; whereas MO of the pterygoid muscle is more debilitating and, thus, the management of these types of patient is more complicated than that of the patients exhibiting MO of other masticatory muscles. According to the experiences of the present study, the following approaches should be considered: i) Transoral and extraoral approaches, which are often used to provide access to the medial aspect of the mandibular ramus to allow complete excision of the ossified muscle; ii) protection of the internal maxillary artery and inferior alveolar nerves (this is considered to be critical); and iii) using a BFP flap to fill the dead space for preventing hematoma formation and heterotopic bone reformation (29,30). Two types of free fat, abdominal fat and the BFP, have been reported that may serve as interpositional material. The BFP has been identified as a particularly effective autogenous tissue, which has been demonstrated in a multitude of surgical procedures in the maxillofacial region (31,32). The BFP lies in close proximity to the site of surgery and may be used as a pedicled or random-pattern flap along with its own blood supply, so there are fewer instances of resorption when compared with an abdominal fat transfer.

In conclusion, a case of PIMO in the medial and lateral pterygoid muscles is presented and chronic low-grade infection was identified to be an important consideration in addition to other possible precipitating factors in the occurrence of MO. Panoramic radiography revealed the source of infection and CT scans effectively delineated the calcified mass. A positive outcome was achieved for the patient by the surgical excision of the calcification and ossified muscles, and via the use of a BFP flap to fill the dead space. This study indicates that symptomatic wisdom teeth must be removed as soon as possible, to prevent infection. In addition, it is important to considered infection as a factor which may lead to myositis ossificans.

## Figures and Tables

**Table I:** Case reports of myositis ossificans in mascatory muscles (20 cases reported since 2001).

Author (Ref.)	Patient (Age, years/Gender)	Location	Chief complaint	History of trauma	Disease duration	MIO (preopertion)	Treatment	MIO (Intraoperation)	MIO (follow-up)	Outcome
Nemoto *et al* (5)	39/M	Masseter; temporalis; lateral pterygoid; frontalis	Trismus mass	Repeatedly struck on the face with a plastic hammer	>1 year	5 mm	Excision + muscle release + bilateral coronoidecomy	55 mm	37 mm (1 year)	No recurrence
Jayade *et al* (2)	25/F	Medial pterygoid; lateral pterygoid; temporalis	Pain; swelling; trismus	None	>6 months	2 mm	Osteotomy + excision + contralateral coronoidectomy	45 mm	39 mm (3 months)	No recurrence
Guarda-Nardini *et al* (4)	50/M	Temporalis	Pain; trismus	Trauma injury	40 days	12 mm	Excision + coronoidectomy	Good	35 mm (6 months)	No recurrence
Choudharya *et al* (6)	31/M	Medial pterygoid	Trismus; mass	Panfacial trauma	3 years	8 mm	Excision	37 mm	27 mm (30 months)	No recurrence
Thangavelu *et al* (11)	36/F	Medial pterygoid;	Trismus; pain	Anesthesia injection + tooth extraction	3 months	3 mm	Osteotomy + excision + abdominal fat graft	32 mm	28 mm (9 months)	No recurrence
Godhi *et al* (19)	21/M	Medial pterygoid; lateral pterygoid; temporalis	Pain; swelling; trismus; mass	None	6 years	5 mm	Osteotomy + excision + reconstruction plate with a condyle	42 mm	Gradual decline (1 year)	Unknown
Trautmann *et al* (12)	33/M	Medial pterygoid	Trismus; tenderness; swelling	Anesthetic injection + endodontic treatment	2 months	5 mm	Coronoidectomy + partial resection of calcified medial pterygoid	Unknown	Limitation (3 years after the second surgery)	Recurred twice
Ramieri *et al* (13)	64/M	Medial pterygoid	Swelling; trismus	Anesthetic injection + tooth extraction	3 years	15 mm	Excision	38 mm	Unknown	Unknown
Kruse *et al* (20)	35/F	Masseter	Trismus; tenderness	None	12 years	10 mm	Conservation treatment	Unknown	Unchanged (10 mm)	Regular follow-up
Conner and Duffy (1)	18/F	Medial pterygoid; temporalis masseter	Pain; trismus;	Anesthetic injection + teeth extraction	4 months	4 mm	Excision + coronoidectomy	Unknown	25 mm (>18 months after the third surgery)	No recurrence (after the third surgery)
Bansal *et al* (16)	20/F	Buccinators; medial pterygoid	Trismus	Dento alveolar trauma	2 years	1 mm	Excision + bilateral coronoidecomy + ipsilateral palatal pedicle flap	35 mm	30 mm (1 year)	No recurrence
Rattan *et al* (18)	45/M	Medial pterygoid	Trismus	Absolute alcohol injection	8 months	7 mm	Excision + pedicled buccal fat pad flap	30 mm	45 mm (2 years)	No recurrence
Mazano *et al* (21)	51/M	Temporalis	Trismus; mass	Severe trauma	25 years	13 mm	Excision	Unknown	38 mm (1 year)	No recurrence
Yano *et al* (8)	34/M	Masseter	Trismus	Criminal violence	Half year	5 mm	Excision + coronoidecomy	30 mm	40 mm (10 months)	No recurrence
Uematsu *et al* (22)	38/F	Temporalis	Pain; mass	None	2 weeks	Unknown	Excision	Unknown	Unknown	Unknown
St Hilaire *et al* (14)	68/M	Masseter; temporalis	trismus	Anesthesia injection + tooth treatment	2 weeks	5 mm	Coronoidectomy + excision + a penrose drain placed	41 mm	40 mm (3.5 years)	No recurrence
Saka *et al* (9)	33/M	Temporalis	Trismus; pain; swelling	Blunt trauma	3 weeks	Limited mouth opening	Excision	Unknown	Unlimited mouth opening (4 years)	No recurrence
Aoki *et al* (10)	44/M	Masseter; lateral pterygoid;	Trismus; pain; swelling; tenderness	Blunt trauma to the face	1 year	7 mm	Excision	32 mm	10 mm (10th day after surgery)	Recurrence
Kim *et al* (15)	30/F	Lateral pterygoid;	Trismus	Anesthesia injection + tooth treatment	3 years	8 mm	Coronoidectomy + excision + interpositional abdominal fat graft placed	27 mm (in the final surgery)	12 mm passively (after the final surgery)	Multiple recurrences
Mevio *et al* (17)	55/F	Temporalis	Trismus	Dental surgery	18 months	6 mm	Coronoidectomy + excision	Unknown	Correct mouth opening	No recurrence

MIO, maximal incisal opening.

**Table II:** Clinical features of myositis ossificans in the masticatory muscles (20 cases reported from 2001) for 12 males and eight females (mean age, 36.75 years).

Parameter	Patients, n
Location	
Masseter	6
Lateral pterygoid	6
Medial pterygoid	11
Temporal	10
Chief complaint	
Trismus	20
Pain	8
Mass	5
Swelling	6
Tenderness	3
Recurrence	
No	13
Yes	4
Unknown	4

**Table III:** Precipitating factors of myositis ossificans in the masticatory muscles (20 cases reported from 2001).

	Patients	
	
Precipitating factor	n	%
Facial trauma	8	40
Local infiltration of anesthetics	6	30
Dental surgery	1	5
Local infiltration of absolute alcohol	1	5
Unknown	4	20
